# TGF-β1/SH2B3 axis regulates anoikis resistance and EMT of lung cancer cells by modulating JAK2/STAT3 and SHP2/Grb2 signaling pathways

**DOI:** 10.1038/s41419-022-04890-x

**Published:** 2022-05-19

**Authors:** Li-Na Wang, Zi-Teng Zhang, Li Wang, Hai-Xiang Wei, Tao Zhang, Li-Ming Zhang, Hang Lin, Heng Zhang, Shao-Qiang Wang

**Affiliations:** 1 Department of Thoracic Surgery, Affiliated Hospital of Jining Medical University, Jining Medical University, 272029 Jining, Shandong Province P. R. China; 2 Medical Research Center, Affiliated Hospital of Jining Medical University, Jining Medical University, 272029 Jining, Shandong Province P. R. China; 3 Department of Thoracic Surgery, The Second Xiangya Hospital of Central South University, 410011 Changsha, Hunan Province P. R. China; 4 Hunan Key Laboratory of Early Diagnosis and Precise Treatment of Lung Cancer, The Second Xiangya Hospital of Central South University, 410011 Changsha, Hunan Province P. R. China; 5 Department of General Thoracic Surgery, Xiangya Hospital, Central South University, 410008 Changsha, Hunan Province P. R. China; 6 Xiangya Lung Cancer Center, Xiangya Hospital, Central South University, 410008 Changsha, Hunan Province P. R. China; 7 Hunan Engineering Research Center for Pulmonary Nodules Precise Diagnosis & Treatment, 410008 Changsha, Hunan Province P. R. China; 8 National Clinical Research Center for Geriatric Disorders (Xiangya Hospital), Changsha, P. R. China

**Keywords:** Cell migration, Lung cancer

## Abstract

The pathogenesis of lung cancer, the most common cancer, is complex and unclear, leading to limited treatment options and poor prognosis. To provide molecular insights into lung cancer development, we investigated the function and underlying mechanism of SH2B3 in the regulation of lung cancer. We indicated SH2B3 was diminished while TGF-β1 was elevated in lung cancer tissues and cells. Low SH2B3 level was correlated with poor prognosis of lung cancer patients. SH2B3 overexpression suppressed cancer cell anoikis resistance, proliferation, migration, invasion, and EMT, while TGF-β1 promoted those processes via reducing SH2B3. SH2B3 bound to JAK2 and SHP2 to repress JAK2/STAT3 and SHP2/Grb2/PI3K/AKT signaling pathways, respectively, resulting in reduced cancer cell anoikis resistance, proliferation, migration, invasion, and EMT. Overexpression of SH2B3 suppressed lung cancer growth and metastasis in vivo. In conclusion, SH2B3 restrained the development of anoikis resistance and EMT of lung cancer cells via suppressing JAK2/STAT3 and SHP2/Grb2/PI3K/AKT signaling cascades, leading to decreased cancer cell proliferation, migration, and invasion.

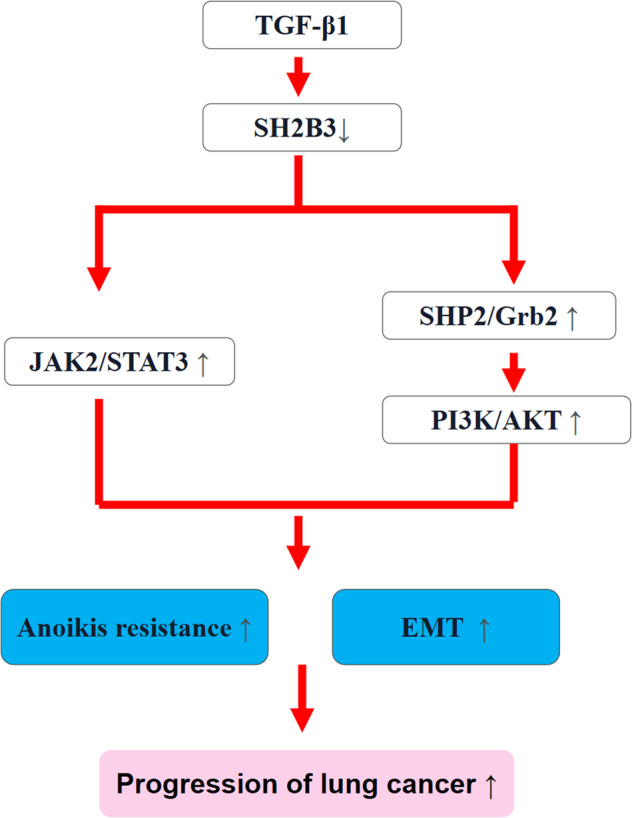

## Introduction

Lung cancer has evolved to become the most prevalent disease and the leading cause of cancer-related death throughout the world [1, 2]. The prognosis of lung cancer is not satisfactory compared to other cancers, and that is primarily due to the complicated and unclear pathogenesis of lung cancer [3].

Anoikis, a programmed cell death, is activated when cells are detached from the extracellular matrix (ECM) [4]. Therefore, anoikis is a crucial mechanism preventing cells from growing in inappropriate or distant locations [4]. The development of anoikis resistance is a crucial way for cancer invasion and metastasis [5]. Epithelial–mesenchymal transition (EMT) program can confer cancer cells to overcome anoikis [6]. Nevertheless, a complete picture of how EMT and anoikis are involved in lung cancer is lacking.

SH2B3 (Src homology 2-b3, also called lymphocyte adapter protein (LNK)) is a member of the family of SH2-containing proteins [7]. Previous studies have indicated that SH2B3 suppresses colorectal carcinoma invasion [8], but facilitates anaplastic thyroid carcinoma growth [9]. However, the study of SH2B3 in lung cancer is limited. Transforming growth factor β1 (TGF-β1) is a secreted cytokine [10], and its role in cancers is complicated in that it could act as both tumor suppressor and promoter, depending on the type and phase of cancers [11]. Previous studies have indicated that TGF-β1 induces EMT to promote lung cancer cell migration and invasion [12]. In lung cancer, TGF-β1 has been shown to facilitate anoikis resistance [12]. However, the molecular mechanism underlying TGF-β1 signaling in modulating SH2B3 expression is not well-understood.

Aberrant activation of Janus kinase 2 (JAK2)/signal transducer and activator of transcription 3 (STAT3) signaling has been reported in the progression of numerous cancers including lung cancer [13]. JAK2/STAT3 signaling can mediate the anoikis resistance of breast cancer cells [14]. Nevertheless, whether JAK2/STAT3 signaling regulates anoikis resistance in lung cancer remains unknown. Moreover, SH2B3 has been shown to negatively regulate JAK2/STAT3 signaling [15]. Whether this regulation exists in lung cancer is not well-understood either.

Src homology region 2-containing protein tyrosine phosphatase 2 (SHP2) has been shown to promote EMT and anoikis resistance during cancer development [16, 17]. One key mechanism is that activated SHP2 recruits Grb2 (growth factor receptor-bound protein 2) to activate downstream signaling, such as the ERK pathway [16, 18, 19]. PI3K/AKT signaling can also be mediated by SHP2 [20]. Nevertheless, whether SH2B3 regulates the SHP2/Grb2 axis is not well-understood.

In this study, we hypothesized that TGF-β1/SH2B3 axis participated in lung cancer development by modulating JAK2/STAT3 and SHP2/Grb2 signaling pathways. Our study elucidated the molecular mechanism of lung cancer progression and provided avenues to develop therapeutic strategies.

## Materials and methods

### Human lung cancer specimen collection

Human lung cancer specimens were taken from 40 diagnosed lung cancer patients during surgery at the Affiliated Hospital of Jining Medical University (Jining, Shangdong, China). The non-tumor tissues near the cancers were collected simultaneously from the same patients. Patients did not receive any preoperative treatments. All patients have consented to the study. The study was reviewed and received approval from the Ethics Committee of the Affiliated Hospital of Jining Medical University. All specimens were put in the liquid nitrogen immediately after collection and then stored in the freezer (−80 °C). The clinicopathological characteristics of lung cancer patients were listed in Supplementary Table 1.

### Cell culture

Six human lung cancer cell lines (A549, NCI-H358, NCI-H1650, NCI-H460, NCI-H1688, Calu-1), one human non-tumorigenic lung epithelial cell line (BEAS-2B), and HEK-293T cells were purchased from Cell Bank of Chinese Academy of Sciences (Shanghai, China). All the cell lines included in this study have been authenticated by STR profiling and tested for mycoplasma contamination. The cells were seeded and grown in Bronchial Epithelial Cell Growth Medium (BEGM, Lonza, Switzerland) containing 10% fetal bovine serum (FBS, Thermo Fisher Scientific, MA, USA) and 1% penicillin–streptomycin (Thermo Fisher Scientific). HEK-293T cells were cultured in Dulbecco’s modified eagle medium (DMEM, Thermo Fisher Scientific) supplemented with 10% FBS and 1% penicillin–streptomycin. The cells were cultured in the CO_2_ incubator at 37 °C. For drug treatment, TGF-β1 (5 ng/mL, Sigma-Aldrich, CA, USA) and AG490 (25 μM, Sigma-Aldrich) were added to the culture medium to stimulate cancer cells.

### Plasmids, cell transfection, and lentivirus

sh-SHP2 and its negative control sh-NC were synthesized from Genepharma (Shanghai, China). Lipofectamine 3000 (Invitrogen, CA, USA) was utilized as the reagent for cell transfection. Briefly, cells were cultured up to 70–80% confluence and then added with the plasmid together with Lipofectamine 3000 at the ratio of 1:1 for 48 h. Sequences for sh-SHP2 were used in this study:

AATTGAGATGTCATTGAGCTTAAATATTCAAGAGATATTTAAGCTCAATGACATCTTTTTTT-sense

GATCAAAAAAAGATGTCATTGAGCTTAAATATCTCTTGAATATTTAAGCTCAATGACATCTC-antisense

SH2B3 full-length, sh-SH2B3 and their negative controls were cloned into the lentivirus vector pLV-CMV. The constructs were transfected into the HEK-293T cells with helper vectors pSPAX2 and pMD2G to generate lentivirus. The lentivirus was used to infect cancer cells for 48 h.

### Co-immunoprecipitation (Co-IP)

Flag-SH2B3 and Myc-JAK2/SHP2 plasmids were all designed and constructed from GeneChem Co., Ltd (Shanghai, China). Lung cancer cells transfected with Flag-SH2B3 and Myc-JAK2/SHP2 or naive lung cancer cells were lysed with lysis buffer supplemented with protease inhibitor. Protein concentration was quantified with Pierce BCA protein Assay (Thermo Fisher Scientific). An equal amount of proteins were incubated with anti-Flag antibody (Thermo Fisher Scientific, cat #MA1-91878, 1:500) or anti-SH2B3 antibody (Santa Cruz Biotechnology, cat #sc-393709, 1:100) for 1 h at 4 °C and then incubated with Protein-A Sepharose beads (Abcam, Cambridge, UK) overnight at 4 °C. The beads were then washed with lysis buffer followed by elution with sodium dodecyl sulfate (SDS) loading buffer. The elution was performed with standard western blot assay.

### Anoikis-resistance assay

Transfected cells were detached from the culture plate and resuspended into single-cell suspension in culture medium, followed by seeding onto the 24-well plates that were pre-coated with poly-HEMA at a density of 1 × 10^5^ cells/mL. After 48 h, the cells were harvested and anoikis was measured by flow cytometry using the Annexin V-FITC/propidium iodide Kit (Thermo Fisher Scientific).

### Soft agar colony-formation assay

The bottom of the six-well plates was pre-coated with 0.6% agarose gel (Sigma-Aldrich). Transfected lung cancer cells were mixed with 0.6% agarose and the cell-agarose mixture was planted onto the agarose gel. The culture plates were put back in the incubator for 2 weeks. Colonies were imaged with a microscope (Olympus, Tokyo, Japan) and the number of colonies was quantified by ImageJ software. The counting process was conducted by an assessor blind to treatment allocation.

### Cell counting kit-8 (CCK-8)

Cell proliferation was measured using the standard CCK-8 Kit (Abcam) according to the manufacturer’s instructions. Cells were plated in the 96-well plates and cultured in the incubator. In total, 10 µL of CCK-8 solution was added to each well and incubated at 37 °C for 2 h. The absorbance at 450 nm was analyzed with the standard microplate reader (BioTek, Winsky, Vermont, USA).

### Scratch wound-healing assay

Cells were seeded in six-well plates and cultured to about 95% confluence. The pipette tip (10 μL) was utilized to make a scratch in the middle of the dish. Cells were rinsed with PBS to wash off dead cells and culture medium without FBS was added back. Images were taken at the time of scratching and 24 h later. Migration distances were analyzed using the ImageJ software and the migration rates were counted as previously described [21]. The counting process was conducted by an assessor blind to treatment allocation.

### Transwell assay

Transfected lung cancer cells were cultured in the culture medium without any serum on top of the filter membrane (8-μm pore) that was pre-coated with Matrigel (Corning, NY, USA) in the transwell insert. A full culture medium that contains 10% FBS was put in the lower chamber. After 24 h, the upper filter was discarded. Cells residing in the lower dish were cells that invaded from the top. Invaded cells were fixed with 4% paraformaldehyde (PFA) first for 10–15 min, and then 0.2% crystal violet was added to stain the cells followed by imaging. The counting process was conducted by an assessor blind to treatment allocation.

### Immunostaining

The culture medium was discarded followed by washing with PBS. In total, 4% PFA was added to fix the cells by incubation for 15 min at room temperature. PFA was washed out by PBS and 0.5% Triton X-100 in PBS was used to permeabilize the cells for 3–5 min at room temperature. Blocking buffer (3% BSA in PBS) was added to block the cells for 1 h at room temperature. Specific primary antibodies including anti-E-cadherin (Cell Signaling Technology, cat #14472, 1:200) and anti-N-cadherin (Cell Signaling Technology, cat #13116, 1:500) were added in the blocking buffer and incubated with cells overnight at 4 °C. Primary antibodies were washed out with PBS three times, followed by incubated with Alexa fluor-conjugated secondary antibodies for 2 h at room temperature. Cells were washed again by PBS three times and then mounted on the slides with DAPI-containing mounting medium (DAPI-Fluoromount-G, Thermo Fisher Scientific). Fluorescence images were captured with a microscope (Olympus, Tokyo, Japan). The accessing process was conducted by an assessor blind to treatment allocation.

### Nude mouse xenograft model

All animal experiments have been reviewed and received approval by the Animal Care and Use Committee of the Affiliated Hospital of Jining Medical University (Jining, Shangdong, China). In total, 120 female BALB/c nude (nu/nu) (8-week-old) were purchased from SJA Laboratory Animal Co., Ltd. (Hunan, China) and raised in the standard animal facility room to randomly divide into four groups. Lung cancer cells were infected with sh-NC, sh-SH2B3, OE-NC, or OE-SH2B3 lentiviruses for 24 h and then unilaterally, subaxillary, and subcutaneously injected into the mice (1 × 10^7^ cells per mouse) to induce tumors. Tumors were monitored every seven days for 28 days. Tumor length (L) and width (W) were analyzed to quantify the tumor volume (V):V (mm^3^) = 0.5 × (W)^2^ × (L). In the end, tumors were harvested for measuring weight and for western blot and immunohistochemistry analyses. The accessing process was conducted by an assessor blind to treatment allocation.

To evaluate lung cancer metastasis, control lung cancer cells or transfected cancer cells (sh-NC, sh-SH2B3, OE-NC, or OE-SH2B3; 5 × 10^5^ cells/100 µL per mouse) were injected into the mice through tail vein injection. 30 days later the animals were sacrificed to harvest the liver tissues for H&E staining to study the liver metastasis. The accessing process was conducted by an assessor blind to treatment allocation.

### Hematoxylin and eosin (H&E) staining

Liver tissues were fixed in 4% PFA buffer overnight at 4 °C and subsequently embedded in paraffin. The liver tissues were then sliced into 5-μm-thick slices and stained with Hematoxylin and Eosin Stain Kit (Vector Labs) as the manufacturer’s instruction described. The accessing process was conducted by an assessor blind to treatment allocation.

### Immunohistochemistry (IHC)

The tumor tissues were dissected out and fixed in 4% PFA at 4 °C overnight, washed by PBS, and then embedded in paraffin. The tumor tissues were sliced into 5-μm-thick sections and dried overnight at 37 °C on glass slides. The dried slices were deparaffinized in xylene first followed by rehydration through a graded concentration of alcohol. 3% hydrogen peroxide was used to quench the sections and 5% bovine serum albumin (BSA) was added to block the slices for 1 h at room temperature. Primary antibodies were added to incubate with the slices at 4 °C overnight. The next day, the antibodies were washed off and secondary antibodies were added to incubate with sections for 2 h at room temperature. Substrates of the Envision system-HRP from the kit (Abcam) were added to incubate with stained slices as the manufacturer’s protocol described and the signals were analyzed with a light microscope (Olympus, Tokyo, Japan). The following primary antibodies were used: anti-SH2B3 (Thermo Fisher Scientific, cat #MA5-25521, 1:50), anti-Ki-67 (Thermo Fisher Scientific, cat #11-5698-82, 1:100), anti-E-cadherin (Cell Signaling Technology, cat #14472, 1:100), and anti-N-cadherin (Cell Signaling Technology, cat #13116, 1:100). The accessing process was conducted by an assessor blind to treatment allocation.

### RNA extraction and qRT-PCR

Trizol (Invitrogen) was employed to extract total RNAs from human lung cancer tissues or cancer cells as the manufacturer’s instruction described. DNaseI was included in the lysis buffer to avoid the contamination of DNA. A commercial cDNA synthesis Kit (SuperScript First-Strand Synthesis System, Thermo Fisher Scientific) was utilized to generate cDNAs through reverse transcription. SYBR Green Master Mix (Invitrogen, China) was used for the quantitative PCR. Relative expression levels of mRNAs were normalized to GAPDH as an internal control. The relative expression levels were calculated by 2^-ΔΔCt^ method. The primers listed as follows were from Genepharma (Shanghai, China):

SH2B3 forward primer: 5’-AGTTCAAGGCCCAAGCTACA-3’;

SH2B3 reverse primer: 5’-AATTCAGCTGCTGCTCGTCT-3’;

GAPDH forward primer: 5’- CTGACTTCAACAGCGACACC-3’;

GAPDH reverse primer: 5’-GTGGTCCAGGGGTCTTACTC-3’.

### Western blot analysis

Proteins from cancer tissues or cells were extracted by utilizing the RIPA lysis buffer (Abcam) according to the standard protocol. DC Protein Assay Kit (Bio-Rad, China) was utilized to quantify the protein concentration. Equal protein from each sample was loaded into sodium dodecyl sulfate (SDS)-polyacrylamide gels and separated through electrophoresis. Later proteins in the gels were transferred to PVDF membranes (Sigma-Aldrich). In all, 3% BSA was used to block the membranes for 30–60 min at room temperature and then specific primary antibodies were added to incubate at 4 °C overnight. The antibodies were discarded and TBST was utilized to wash the membranes three times before incubation with specific secondary antibodies for 1–2 h at room temperature. Protein band intensities were detected by using the Clarity Western ECL substrate kit (Bio-Rad). Primary antibodies used in the study were as follows: anti-SH2B3 (Thermo Fisher Scientific, cat #PA5-52154, 1:2000), anti-MMP-2 (Thermo Fisher Scientific, cat #MA5-13590, 1:1000), anti-MMP-9 (Thermo Fisher Scientific, cat #MA5-15886, 1:2000), anti-E-cadherin (Cell Signaling Technology, cat #3195, 1:1000), anti-N-cadherin (Cell Signaling Technology, cat #4061, 1:1000), anti-slug (Abcam, cat #ab27568, 1:1500), anti-vimentin (Abcam, cat #ab137321, 1:1000), anti-p-JAK2 (Cell Signaling Technology, cat #3771, 1:1500), anti-JAK2 (Cell Signaling Technology, cat #3230, 1:1000), anti-p-STAT3 (Abcam, cat #ab76315, 1:2000), anti-STAT3 (Abcam, cat #ab31370, 1:2000), anti-p-SHP2 (Cell Signaling Technology, cat #3751, 1:1500), anti-SHP2 (Cell Signaling Technology, cat #3752, 1:1500), anti-p-PI3K (Abcam, cat #ab191606, 1:1000), anti-PI3K (Cell Signaling Technology, cat #13666, 1:1000), anti-p-AKT (Cell Signaling Technology, cat #9271, 1:2000), anti-AKT (Cell Signaling Technology, cat #9272, 1:2000), anti-Grb2 (Cell Signaling Technology, cat #3972, 1:1500) and anti-GAPDH (Abcam, cat #ab9485, 1:2000).

### Statistical analysis

All experiments were performed in at least three biological replicates, and each biological replicate contained three technical replicates, and the experimental data were analyzed in GraphPad Prism 7. All the data meet the assumption of normal distribution. The Data were presented as mean ± standard deviation (SD). Statistical details were calculated by Student’s *t* test (for two groups, paired comparison for clinical patient data and unpaired comparison for cell data) or one-way analysis of variance (ANOVA) followed by Tukey’s post hoc test (for groups more than two). The correlation between clinicopathological characteristics of lung cancer patients and SH2B3 expression was assessed by the Chi-squared test. *P* values less than 0.05 were considered statistically significant.

## Results

### SH2B3 was diminished in lung cancer tissues and cells

**Fig. 1:**
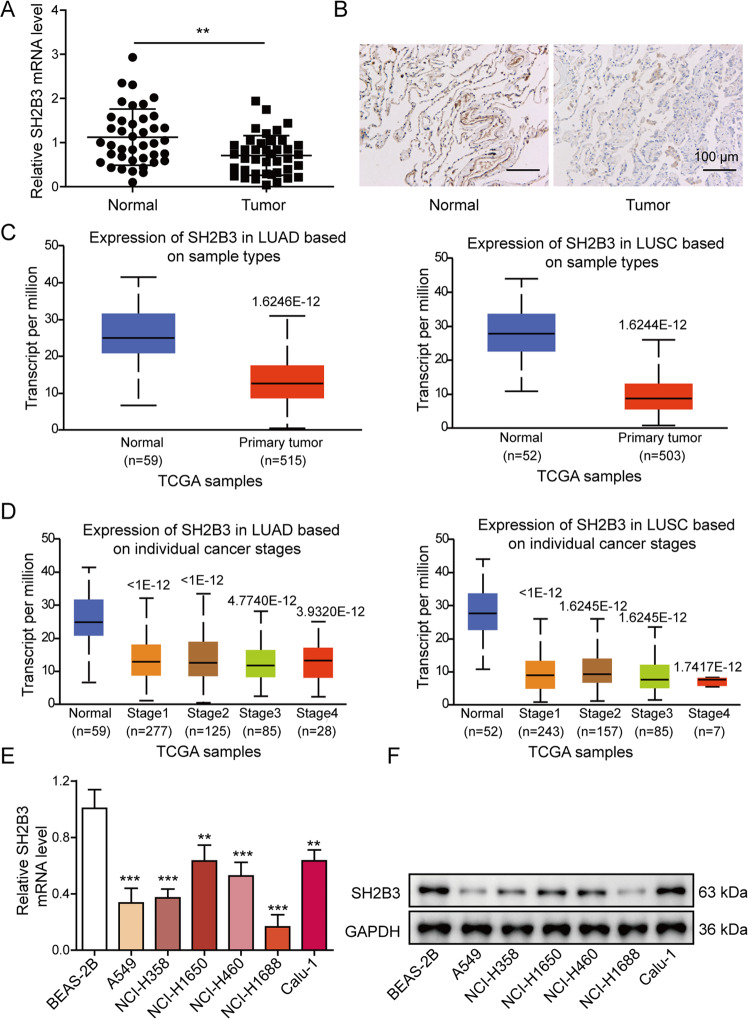
SH2B3 was diminished in lung cancer tissues and cells. **A** qRT-PCR analysis of SH2B3 mRNA levels in human lung cancer tissues (*n* = 40). **B** IHC assay to examine SH2B3 protein levels in human lung cancer tissues. **C** Bioinformatic analysis of SH2B3 mRNA level in human lung cancer tissues from UALCAN database. **D** Bioinformatic analysis of SH2B3 mRNA level in lung cancer tissues from patients at different stages from UALCAN database. **E** qRT-PCR analysis of SH2B3 mRNA levels in cultured lung cancer cell lines. **F** Western blotting to examine SH2B3 protein levels in cultured lung cancer cell lines. ***P < 0.001 and **P < 0.01.

SH2B3 mRNA and protein levels were significantly diminished in 40 diagnosed lung cancer tissues (Fig. 1A, B). Consistently, in UALCAN database, we found that SH2B3 expression was lower in both lung adenocarcinoma (LUAD) and lung squamous cell carcinoma (LUSC) tissues (Fig. 1C). Moreover, analysis from this database indicated that the SH2B3 level was much lower in patients at advanced stages (Fig. 1D). Also, we observed in Supplementary Table 1 that low SH2B3 expression was associated with lymph node metastasis and the TNM stage. We also observed that both mRNA and protein levels of SH2B3 were significantly lower in lung cancer cells (Fig. 1E, F). Given that SH3B3 level is the lowest in A549 and NCI-H1688 cells, we used these two cell lines for subsequent experiments.

### SH2B3 promoted anoikis but suppressed malignant phenotypes of lung cancer

**Fig. 2:**
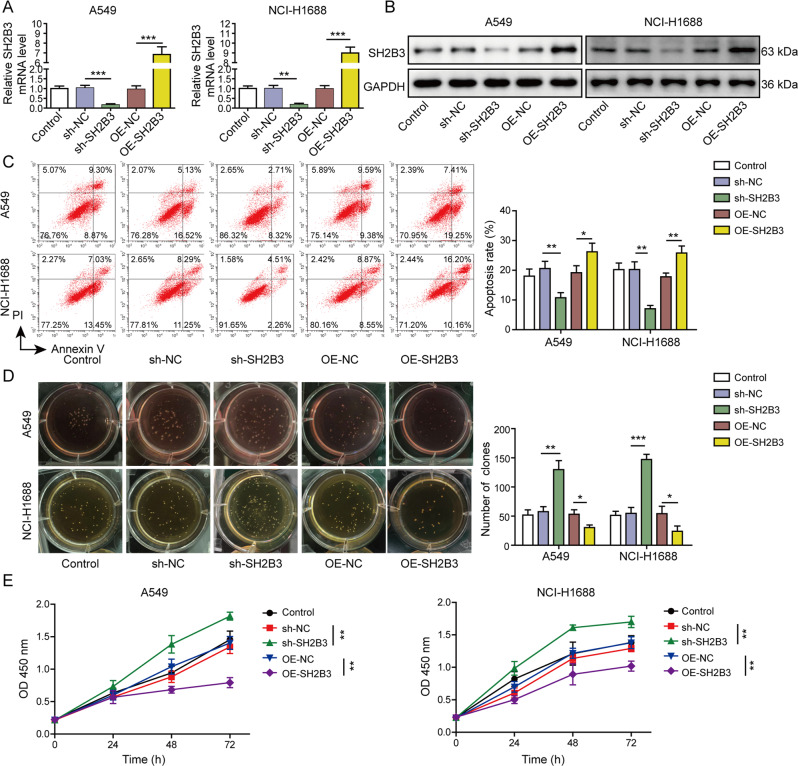
SH2B3 promoted anoikis but suppressed cell proliferation of lung cancer. **A** qRT-PCR analysis of SH2B3 mRNA levels in transfected cells. **B** Western blotting to examine SH2B3 protein levels in transfected cells. **C** Flow cytometry to measure anoikis resistance of transfected cancer cells. **D** Soft colony formation to assess the number of anoikis resistance of cancer cells. **E** CCK-8 assay to evaluate the proliferation rate of transfected cancer cells. ***P < 0.001, **P < 0.01, and *P < 0.05.

**Fig. 3:**
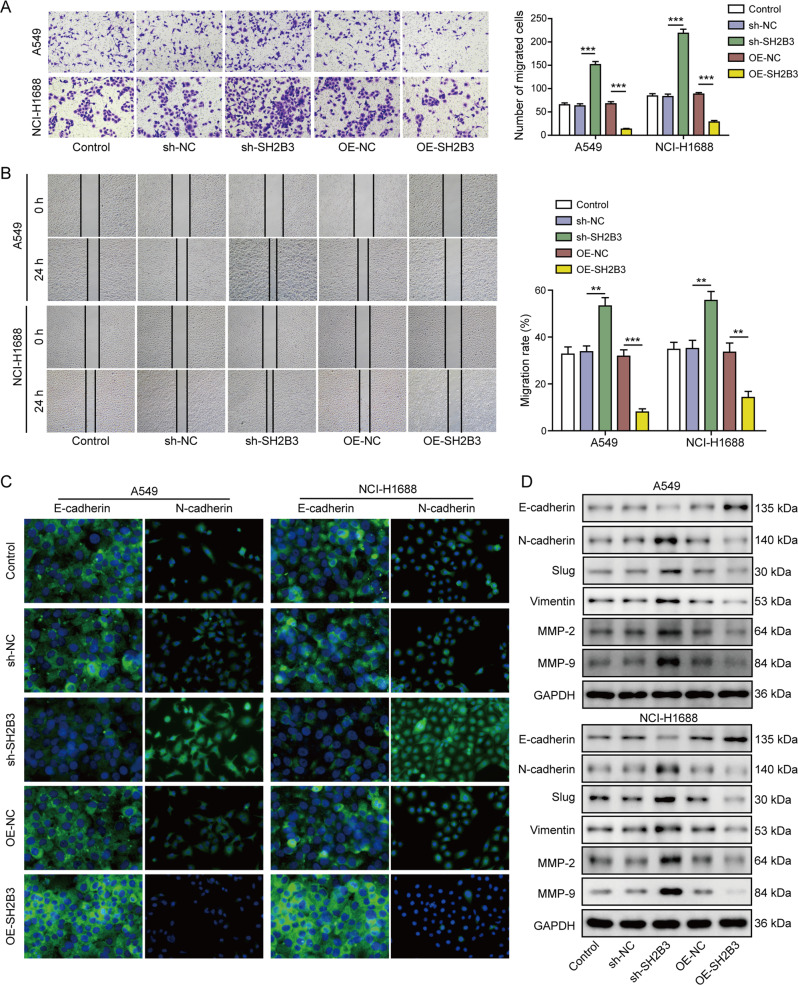
SH2B3 suppressed EMT and cell migration and invasion of lung cancer. **A** Transwell assay to measure invasion capacity of transfected cancer cells. **B** Scratch wound-healing assay to determine the migration ability of transfected cancer cells. **C** Immunostaining to test E-cadherin and N-cadherin protein levels in transfected cancer cells. **D** Western blotting to measure levels of EMT-related markers including E-cadherin, N-cadherin, slug, vimentin, MMP-2, and MMP-9 in transfected cancer cells. ***P < 0.001 and **P < 0.01.

We next investigated how SH2B3 regulated anoikis resistance and malignant phenotypes of lung cancer. The remarkably decreased or upregulated SH2B3 mRNA and protein levels were found after transfection of lung cancer cells with sh-SH2B3 or OE-SH2B3, respectively (Fig. 2A, B). Knockdown of SH2B3 significantly decreased the number of anoikis cells while overexpression of SH2B3 increased (Fig. 2C, D). Also, the knockdown of SH2B3 was greatly enhanced while overexpression of SH2B3 suppressed the proliferation, migration, and invasion of cancer cells (Figs. 2E and 3A, B). Higher E-cadherin signaling but lower N-cadherin signaling was observed in OE-SH2B3-transfected cells, while transfection of sh-SH2B3 exerted the opposite effects (Fig. 3C). Consistently, knockdown of SH2B3 downregulated E-cadherin protein but upregulated protein levels of N-cadherin, slug, vimentin, MMP-2, and MMP-9, and overexpression of SH2B3 induced the opposite changes (Fig. 3D). To further confirm the role of SH2B3 in lung cancer, we repeated the experiments with Calu-1 cells and observed similar effects (Supplemental Fig. 1). Altogether, these data demonstrate that SH2B3 accelerates cancer cell anoikis and inhibits EMT process, cell proliferation, migration, and invasion.

### TGF-β1 regulated lung cancer anoikis and malignant phenotypes via SH2B3

We found that TGF-β1 treatment significantly diminished mRNA and protein levels of SH2B3 in cancer cells while transfection of cells with OE-SH2B3 restored SH2B3 levels (Supplementary Fig. 2A, B). TGF-β1 treatment decreased the number of anoikis cells and enhanced the cancer cell proliferation while overexpression of SH2B3 reversed these changes (Supplementary Fig. 2C, D). TGF-β1 application remarkably increased the migration and invasion of cells (Supplementary Fig. 3A, B). Again, transfection of OE-SH2B3 suppressed those increases (Supplementary Fig. 3A, B). Regarding EMT process, we observed that TGF-β1 treatment upregulated the levels of N-cadherin, slug, vimentin, MMP-2, and MMP-9, but downregulated E-cadherin level (Supplementary Fig. 3C, D). Overexpression of SH2B3 reversed the changes induced by TGF-β1 (Supplementary Fig. 3C, D). In human lung cancer tissues, we found that TGF-β1 was significantly upregulated (Supplementary Fig. 3E). Moreover, from GEPIA database, LUAD and LUSC patients with higher TGF-β1 level survived shorter than patients with lower TGF-β1 level (Supplementary Fig. 3F). These results prove that TGF-β1 suppresses anoikis and facilitates EMT, and proliferation, migration, and invasion of lung cancer via decreasing SH2B3 expression.

### TGF-β1 modulated JAK2/STAT3 and SHP2/Grb2/PI3K/AKT signaling pathways via SH2B3

To investigate the molecular mechanisms underlying the function of the TGF-β1/SH2B3 axis in lung cancer, we next determined whether and how TGF-β1 modulated JAK2/STAT3 and SHP2/Grb2/PI3K/AKT signaling. As shown in Supplementary Fig. 4A, TGF-β1 treatment significantly increased p-JAK2 and p-STAT3 intensities, but overexpression of SH2B3 reversed those increases (Supplementary Fig. 4A). Consistently, TGF-β1 treatment upregulated the protein levels of p-SHP2, Grb2, p-PI3K, and p-AKT while overexpression of SH2B3 reversed the effects of TGF-β1 (Supplementary Fig. 4B). These results show that TGF-β1 activates both JAK2/STAT3 and SHP2/Grb2/PI3K/AKT signaling pathways via SH2B3.

### SH2B3 bound to JAK2 and suppressed JAK2/STAT3 signaling

**Fig. 4:**
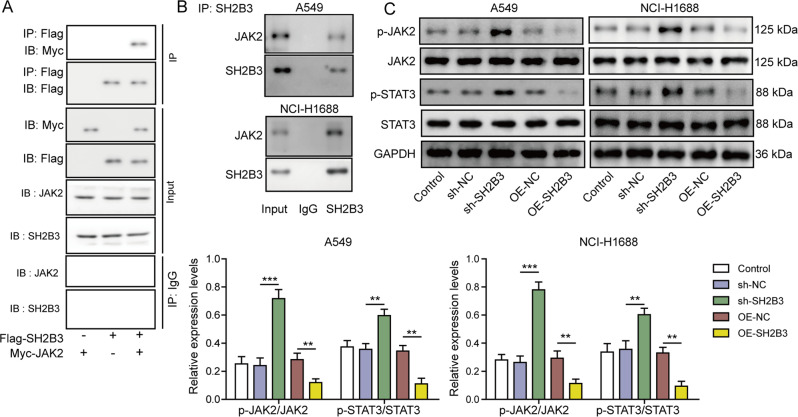
SH2B3 bound to JAK2 and suppressed JAK2/STAT3 signaling. **A** Immunoblotting for Flag, Myc, JAK2, and SH2B3 following immunoprecipitation with Flag antibody in transfected cells. **B** Immunoblotting for JAK2 following SH2B3 immunoprecipitation in cancer cells. **C** Western blotting to determine protein levels of p-JAK2, JAK2, p-STAT3, and STAT3 in transfected cancer cells. ***P < 0.001 and **P < 0.01.

To study how SH2B3 regulates JAK2/STAT3 signaling, immunoprecipitation with Flag antibody successfully pulled down Myc-JAK2 when Flag-SH2B3 and Myc-JAK2 were co-transfected into cells together (Fig. 4A). Furthermore, with a specific SH2B3 antibody, we detected JAK2 expression following SH2B3 immunoprecipitation in lung cancer cells (Fig. 4B). These results indicated that SH2B3 bound to JAK2. In cells transfected with sh-SH2B3, we observed higher levels of p-JAK2 and p-STAT3 (Fig. 4C). In contrast, overexpression of SH2B3 diminished those expression levels (Fig. 4C). We thus conclude that SH2B3 suppresses JAK2/STAT3 signaling by interacting with JAK2.

### SH2B3 regulated anoikis resistance and EMT via JAK2/STAT3 signaling

Next, we determined the roles of SH2B3/JAK2 interaction in lung cancer. As expected, transfection of cells with sh-SH2B3 diminished SH2B3 expression level but increased p-JAK2 and p-STAT3 expression levels (Supplementary Fig. 5A). The application of AG490, a specific and potent JAK2 inhibitor, downregulated the p-JAK2 and p-STAT3 expression levels but did not affect SH2B3 expression (Supplementary Fig. 5A). Knockdown of SH2B3 decreased the percentage of anoikis cells, but increased the proliferation, migration, and invasion abilities of cancer cells (Supplementary Figs. 5B–D and 6A, B). Notably, AG490 treatment suppressed all those effects mediated by sh-SH2B3 (Supplementary Figs. 5B–D and 6A, B). Regarding EMT process, cells transfected with sh-SH2B3 had a lower level of E-cadherin and higher levels of N-cadherin, slug, vimentin, MMP-2, and MMP-9 (Supplementary Fig. 6C, D). The application of AG490 to sh-SH2B3-transfected cells reversed those changes (Supplementary Fig. 6C, D). Taken together, our results demonstrate that SH2B3 modulates anoikis, EMT, and proliferation, migration, and invasion of lung cancer cells by suppressing JAK2/STAT3 signaling.

### SH2B3 bound SHP2 to inhibit SHP2/Grb2/PI3K/AKT signaling

**Fig. 5:**
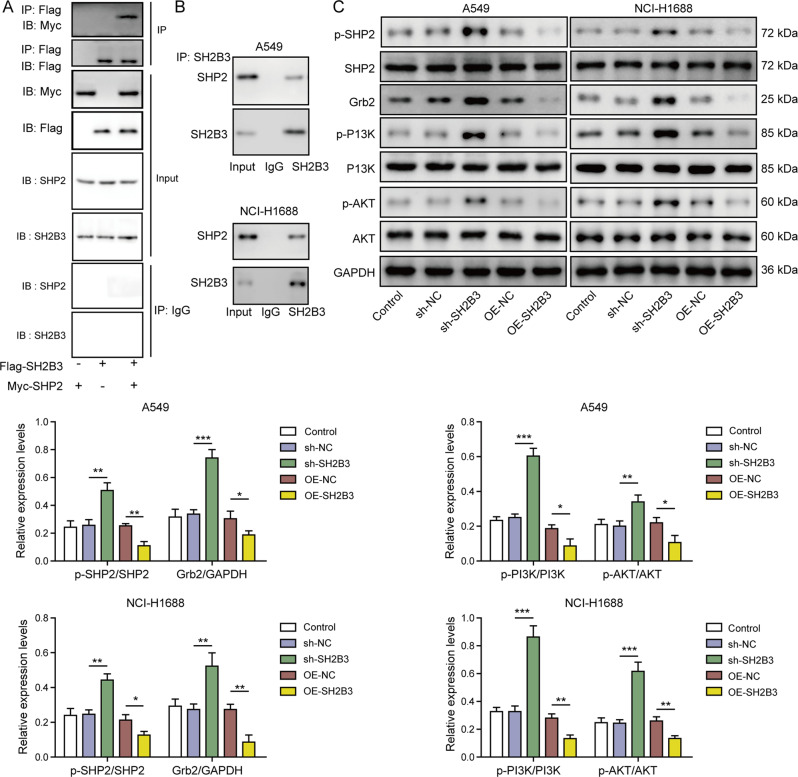
SH2B3 bound to SHP2 to inhibit SHP2/Grb2/PI3K/AKT signaling. **A** Immunoblotting for Flag, Myc, SHP2, and SH2B3 following immunoprecipitation with Flag antibody in transfected cells. **B** Immunoblotting for SHP2 following SH2B3 immunoprecipitation in cancer cells. **C** Western blotting to determine protein levels of p-SHP2, SHP2, Grb2, p-PI3K, PI3K, p-AKT, and AKT in transfected cancer cells. ***P < 0.001, **P < 0.01, and *P < 0.05.

To investigate how SH2B3 affects SHP2/Grb2/PI3K/AKT signaling, we again examined whether SH2B3 interacted with SHP2. We found that immunoprecipitation with Flag antibody pulled down Myc-SHP2 (Fig. 5A). Consistently, immunoprecipitation with SH2B3 antibody pulled down endogenous SHP2 in lung cancer cells (Fig. 5B). These results indicated that SH2B3 binds with SHP2. In cells transfected with sh-SH2B3, we observed elevated levels of p-SHP2, Grb2, p-PI3K, and p-AKT (Fig. 5C). In contrast, cells transfected with OE-SH2B3 exhibited lower levels (Fig. 5C). Together, these data show that SH2B3 binds to SHP2 to inhibit the SHP2/Grb2/PI3K/AKT signaling pathway.

### SH2B3 regulated anoikis and EMT via SHP2/Grb2/PI3K/AKT signaling

We then studied the function of SH2B2/SHP2 interaction in lung cancer. As shown in the above results, knockdown of SH2B3 upregulated the levels of p-SHP2, Grb2, p-PI3K, and p-AKT, while co-transfection of sh-SHP2 and sh-SH2B3 downregulated those levels (Supplementary Fig. 7A). Again, cells transfected with sh-SH2B3 alone had a lower percentage of anoikis cells, and a higher proliferation rate, as well as enhanced migration and invasion abilities (Supplementary Figs. 7B–D and 8A, B). However, knockdown of SHP2 suppressed those effects caused by sh-SH2B3 transfection (Supplementary Figs. 7B–D and 8A, B). Moreover, sh-SH2B3 promoted EMT process while sh-SHP2 reversed those changes (Supplementary Fig. 8C, D). Altogether, these results prove that SH2B3 inhibits anoikis resistance, EMT, cell proliferation, migration, and invasion of lung cancer via suppressing SHP2/Grb2/PI3K/AKT signaling.

### SH2B3 restrained lung cancer growth and metastasis in vivo

**Fig. 6:**
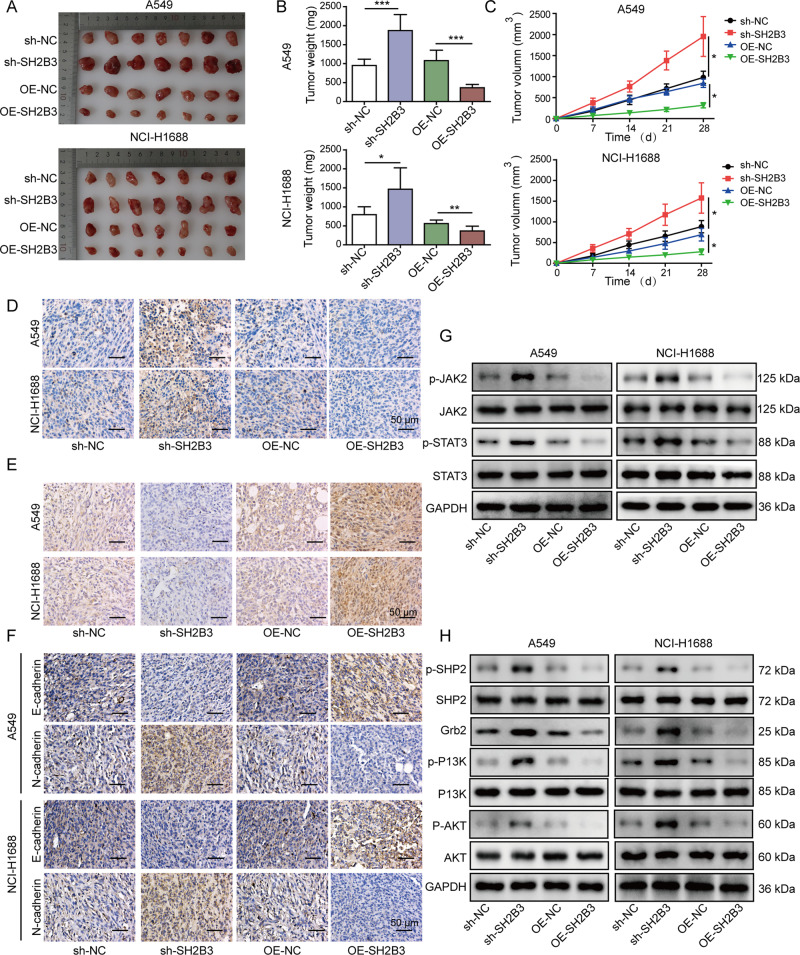
SH2B3 restrained lung cancer growth in vivo. **A** Representative tumor images from each group of mice. **B**, **C** Quantification of tumor weight (**B**) and size (**C**) in each group of mice after 28 days of implantation. **D** IHC to examine Ki-67 level in tumor samples from each group of mice. **E** IHC to test SH2B3 level in tumor samples from each group of mice. **F** IHC to evaluate N-cadherin and E-cadherin expression levels in tumor samples from each group of mice. **G** Western blotting to determine p-JAK2, JAK2, p-STAT3, and STAT3 in tumor samples from each group of mice. **H** Western blotting to determine p-SHP2, SHP2, Grb2, p-PI3K, PI3K, p-AKT, and AKT in tumor samples from each group of mice. ***P < 0.001, **P < 0.01, and *P < 0.05.

In the end, we evaluated the role of SH2B3 in lung cancer in vivo via using the nude mouse xenograft model. As expected, the tumors from the sh-SH2B3 group were significantly bigger and heavier (Fig. 6A–C). In contrast, overexpression of SH2B3 in tumor cells remarkably decreased the tumor size and weight (Fig. 6A–C). Consistently, Ki-67 signal was strongest in the sh-SH2B3 group, but was the weakest in the OE-SH2B3 group (Fig. 6D). We observed a lower level of SH2B3 in tumors of sh-SH2B3 group but a higher level of SH2B3 in tumors of OE-SH2B3 group (Fig. 6E). In addition, we observed a reduced level of E-cadherin but an increased level of N-cadherin in sh-SH2B3 group (Fig. 6F). OE-SH2B3 group exhibited opposite changes with decreased N-cadherin and elevated E-cadherin (Fig. 6F). With western blotting, we found that the levels of p-JAK2, p-STAT3, p-SHP2, Grb2, p-PI3K, and p-AKT were elevated in the tumors from sh-SH2B3 group, but were diminished in the OE-SH2B3 group (Fig. 6G, H). Taken together, we make the conclusion that SH2B3 restrains lung tumor growth in vivo.

**Fig. 7:**
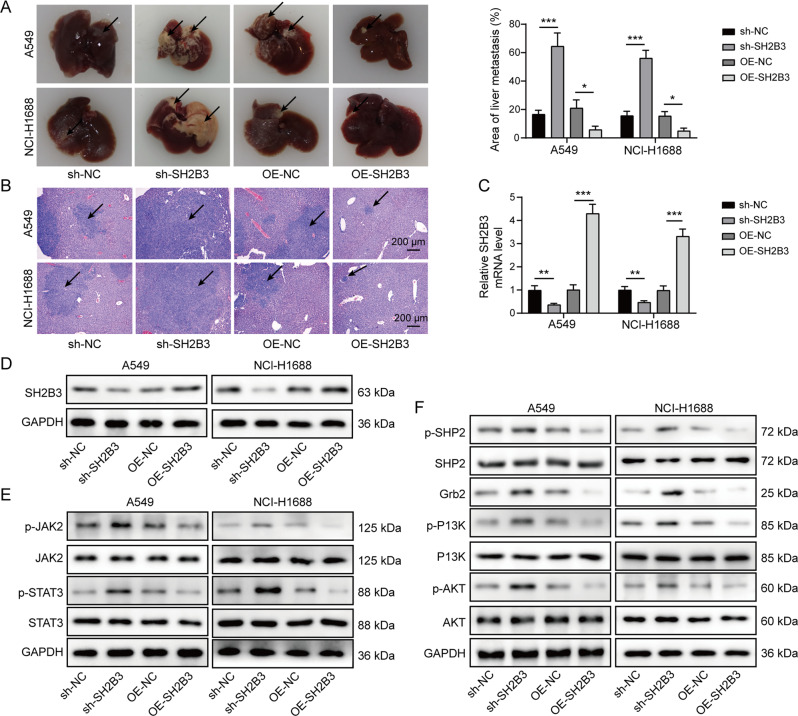
SH2B3 inhibited lung cancer metastasis in vivo. **A** The area of tumor nodules in the liver of each group. **B** H&E staining of metastatic lesions in the liver of each group. **C** qRT-PCR analysis of SH2B3 mRNA levels in the liver tissues of each group. **D** Western blotting to measure SH2B3 protein levels in the liver tissues of each group. **E** Western blotting to determine p-JAK2, JAK2, p-STAT3, and STAT3 in the liver tissues from each group of mice. **F** Western blotting to determine p-SHP2, SHP2, Grb2, p-PI3K, PI3K, p-AKT, and AKT in the liver tissues from each group of mice. ***P < 0.001 and **P < 0.01.

Knockdown of SH2B3 in cancer cells significantly increased the area of tumor nodules in the liver while overexpression of SH2B3 greatly diminished the tumor nodules (Fig. 7A). We observed more and bigger metastatic foci in the sh-SH2B3 group, while there were fewer and smaller metastatic foci in the OE-SH2B3 group (Fig. 7B). We confirmed that sh-SH2B3 decreased SH2B3 mRNA and protein levels in liver tissues while OE-SH2B3 increased these expression levels (Fig. 7C, D). In addition, we showed that knockdown of SH2B3 increased p-JAK2, p-STAT3, p-SHP2, Grb2, p-PI3K, and p-AKT expression levels while overexpression of SH2B3 downregulated those expression levels (Fig. 7E, F). Therefore, we conclude that SH2B3 inhibits lung cancer metastasis in vivo.

## Discussion

Here, through a combination of in vitro and in vivo methods, we fully elucidated that SH2B3 bound to JAK2 and SHP2 to suppress JAK2/STAT3 and SHP2/Grb2/PI3K/AKT signaling pathways respectively, resulting in inhibition on anoikis resistance, EMT, and cancer cell proliferation, migration, and invasion. These findings suggest that targeting SH2B3 could be a potential way to treat lung cancer.

Most of the studies have focused on the role of SH2B3 in hematopoiesis [22, 23]. Recently, emerging evidence suggests that SH2B3 is also involved in cancer development [24, 25]. For instance, SH2B3 is downregulated and overexpression of SH2B3 reduces cancer cell invasion in colorectal carcinoma [8]. In this study, we prove that SH2B3 acts as a tumor suppressor in lung cancer, and to our knowledge, this is the first study to examine the expression and function of SH2B3 in anoikis resistance and EMT of lung cancer. We also have demonstrated that overexpression of SH2B3 greatly suppressed lung cancer growth and liver metastasis in vivo. Previous studies implied a promotive role of SH2B3 in the development of breast cancer and ovarian cancer [25, 26], suggesting a context-dependent function of SH2B3. TGF-β1 signaling has been shown to play a crucial role in the EMT process, but the underlying molecular mechanisms are complex [27]. Here, we provide evidence that SH2B3 is an important downstream effector of TGF-β1 signaling and that SH2B3 overexpression blocks TGF-β1-mediated oncogenic effects. It will be interesting to examine how TGF-β1 regulates SH2B3 expression in the future. Also, the in vivo animal results about the TGF-β1/SH2B3 axis in the regulation of lung cancer development and metastasis will strengthen our manuscript.

Indeed, activation of the JAK2/STAT3 signaling pathway has been reported in various types of cancers [28–30]. Consistently, here we observe that TGF-β1 activates JAK2/STAT3 signaling in lung cancer cells, and overexpression of SH2B3 inhibits this activation. This activation of the JAK2/STAT3 pathway promotes anoikis resistance and EMT, resulting in enhanced malignant phenotypes. Moreover, we show that SH2B3 binds to JAK2 to inhibit JAK2/STAT3 signaling, supporting that hyperactivity of JAK2/STAT3 signaling in lung cancer cells results from diminished SH2B3 expression level. Similar regulation has been reported in other types of cancers [22, 31]. Our study reports for the first time that SH2B3 regulates EMT and anoikis resistance by inhibiting JAK2/STAT3 signaling in lung cancer.

Besides JAK2/STAT3 signaling, we also demonstrate that SH2B3 interacts with SHP2 to inhibit the SHP2/Grb2/PI3K/AKT signaling pathway in lung cancer cells. SHP2 binds with the adaptor protein Grb2 to regulate PI3K/AKT signaling pathway [32]. A handful of studies have shown that the promotion of SHP2/Grb2 interaction is associated with cancer development [16, 33]. Similarly, Grb2-mediated PI3K signaling cascade plays a critical role in the proliferation and migration of cancer cells [33–35]. Consistently, we observe that TGF-β1 promotes the activation of the SHP2/Grb2 axis in lung cancer cells. SH2B3 is an adaptor that binds to many proteins, particularly phosphorylated tyrosine [36]. Here, we show that it can bind to SHP2 as well. Future studies are necessary to examine whether there are other interactors of SH2B3, which mediate the diverse functions of SH2B3. To our knowledge, this work is the first to show that SH2B3 suppresses EMT and anoikis resistance via suppressing SHP2/Grb2/PI3K/AKT signaling.

In summary, our study is the first to illustrate the crucial roles of SH2B3 in lung cancer and reveals that TGF-β1/SH2B3 axis regulates the anoikis resistance and EMT process of lung cancer cells via JAK2/STAT3 and SHP2/Grb2/PI3K/AKT signaling pathways. These findings provide potential targets for the development of future therapy in lung cancer.

## Supplementary information

Supplementary Fig.1

Supplementary Fig.2

Supplementary Fig.3

Supplementary figure legends

Supplementary table 1

Supplementary Fig.4

Supplementary Fig.5

Supplementary Fig.6

Supplementary Fig.7

Supplementary Fig.8

Reproducibility checklist

## Data Availability

All data generated or analyzed during this study are included in this published article.
