# Supplementary figures and images for: TGF-β1/SH2B3 axis regulates anoikis resistance and EMT of lung cancer cells by modulating JAK2/STAT3 and SHP2/Grb2 signaling pathways
Source: Cell Death Dis. 2022 May 19;13(5):472. doi: 10.1038/s41419-022-04890-x (PMC9120066; doi:10.1038/s41419-022-04890-x)

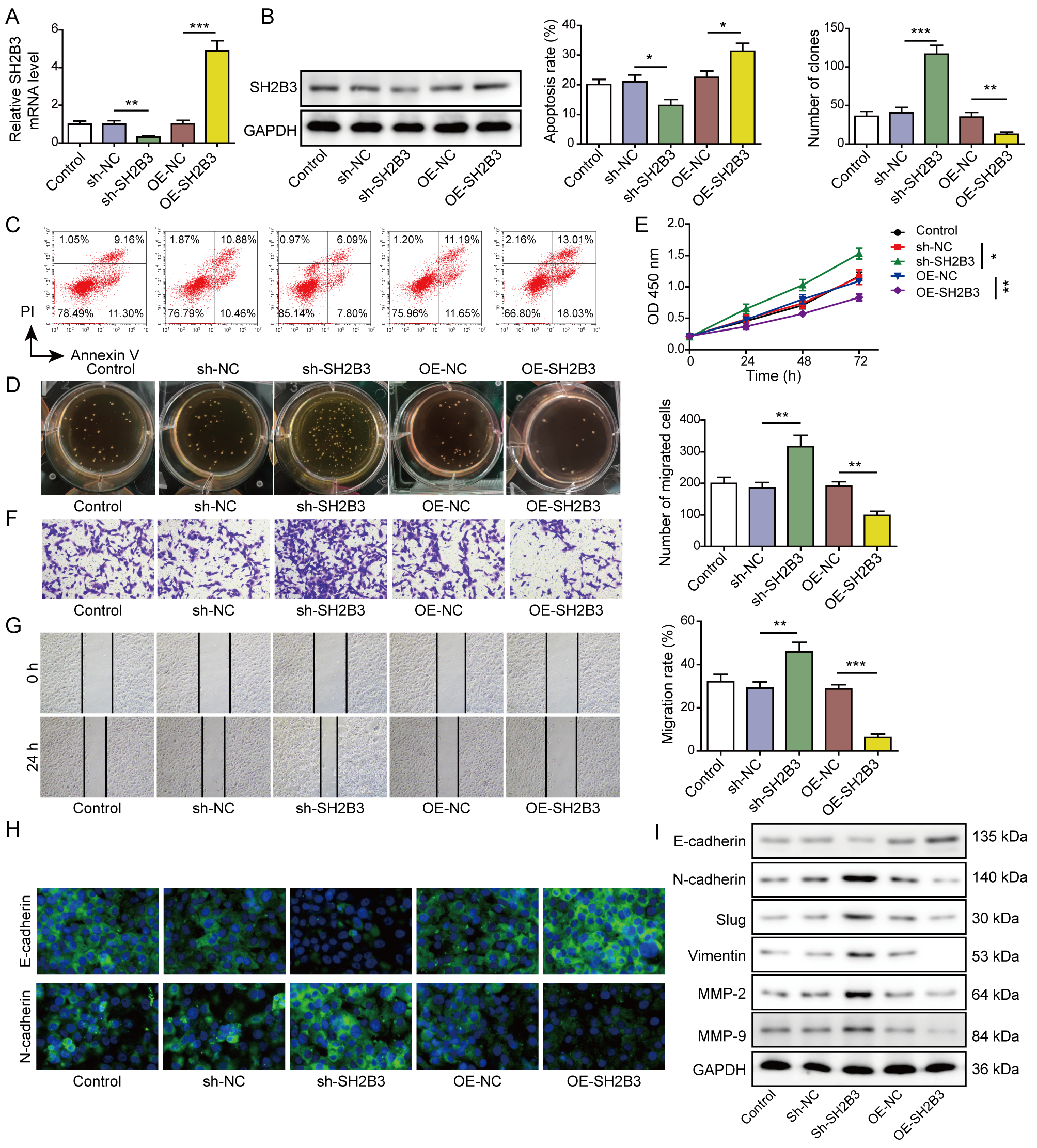

Supplement: Supplementary file 1 — Supplementary Fig.1 [file 41419_2022_4890_MOESM1_ESM.tif]

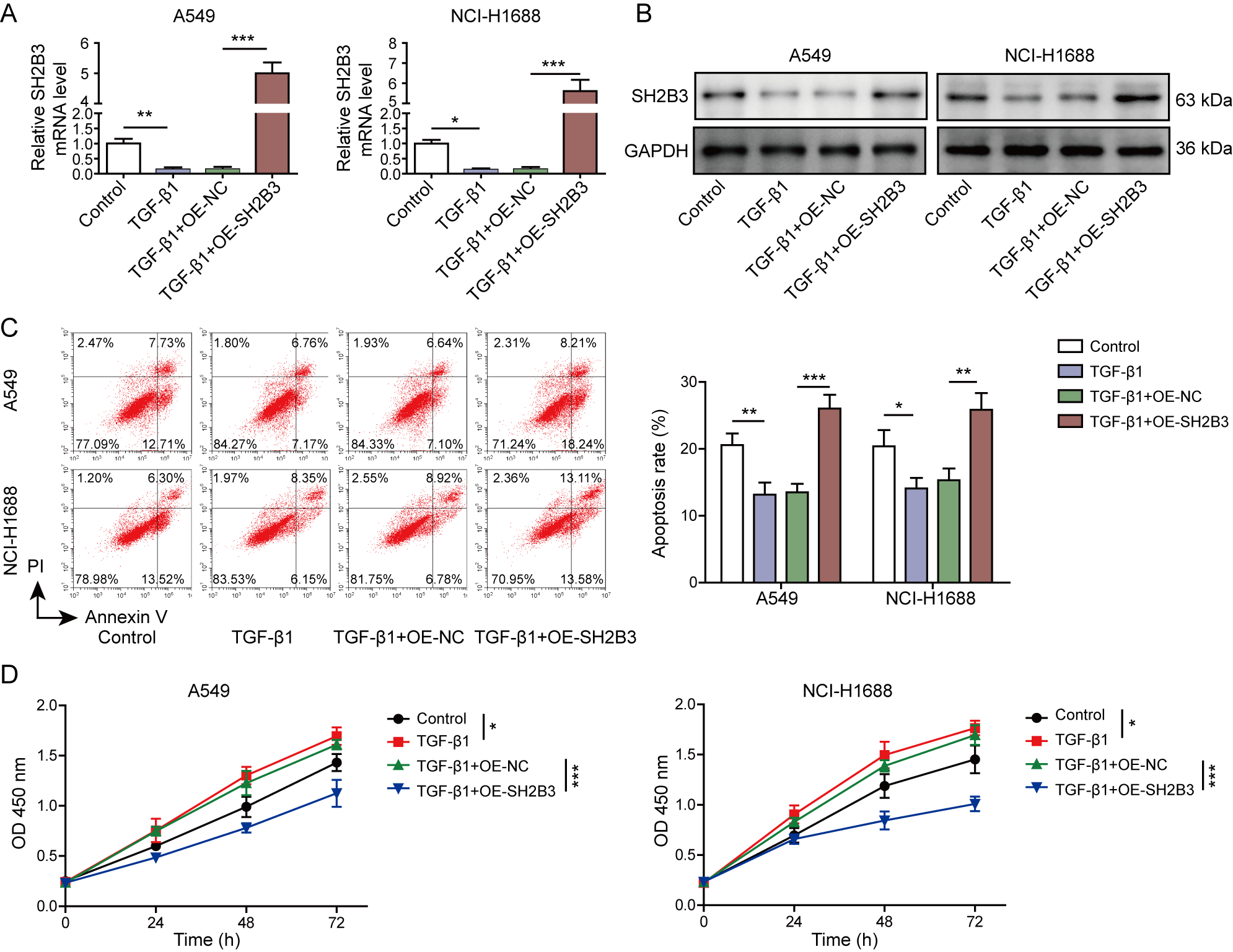

Supplement: Supplementary file 2 — Supplementary Fig.2 [file 41419_2022_4890_MOESM2_ESM.tif]

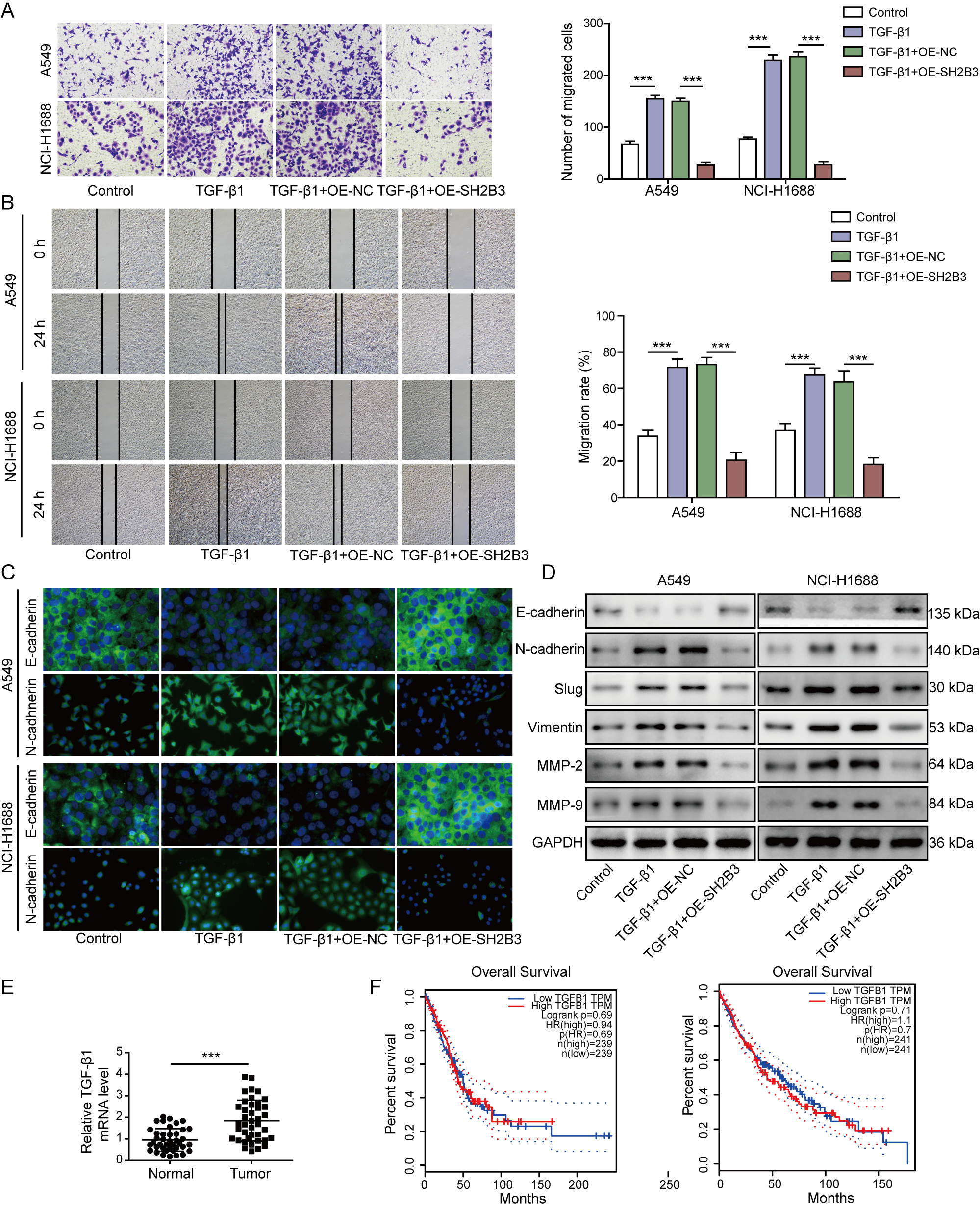

Supplement: Supplementary file 3 — Supplementary Fig.3 [file 41419_2022_4890_MOESM3_ESM.tif]

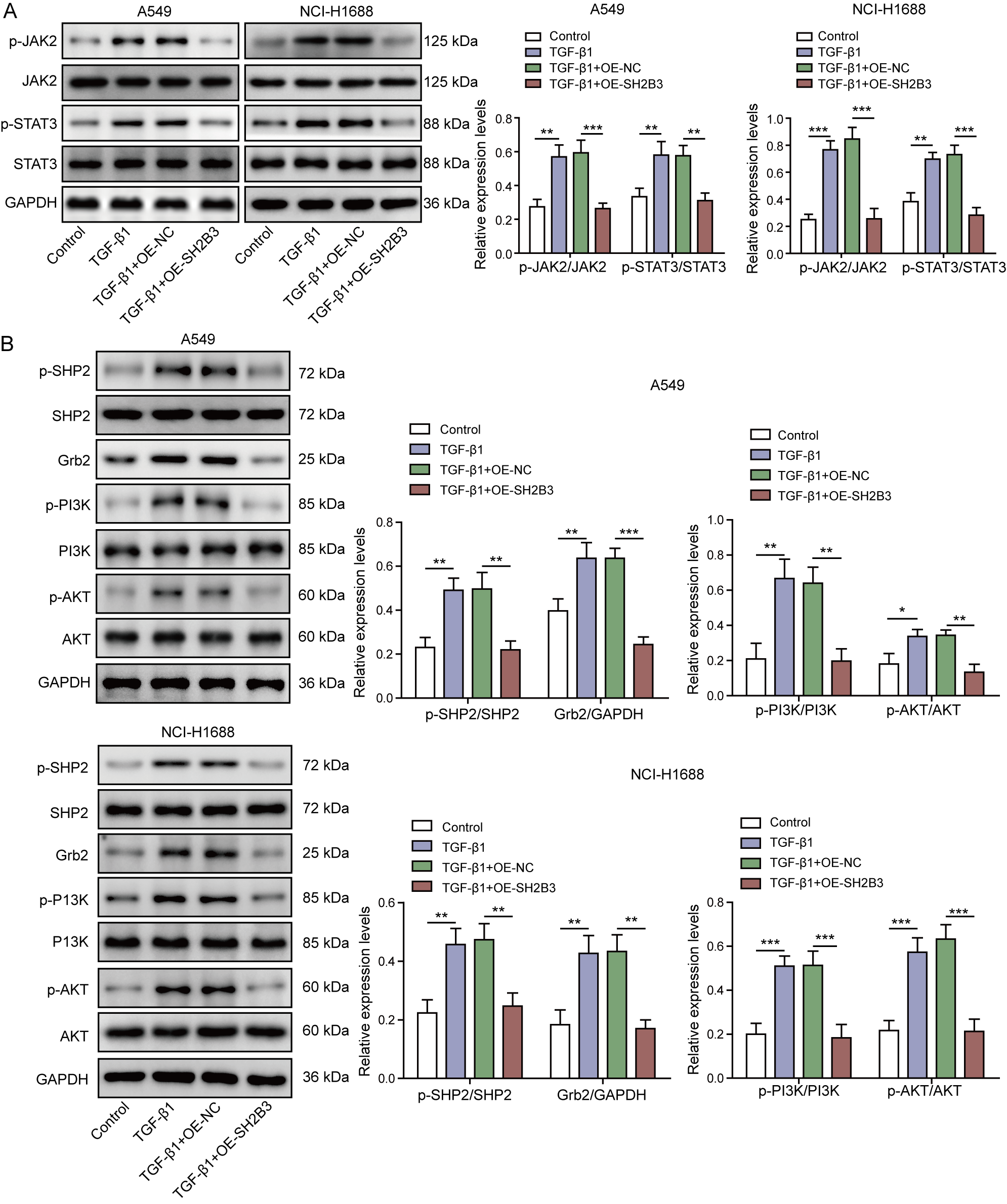

Supplement: Supplementary file 6 — Supplementary Fig.4 [file 41419_2022_4890_MOESM6_ESM.tif]

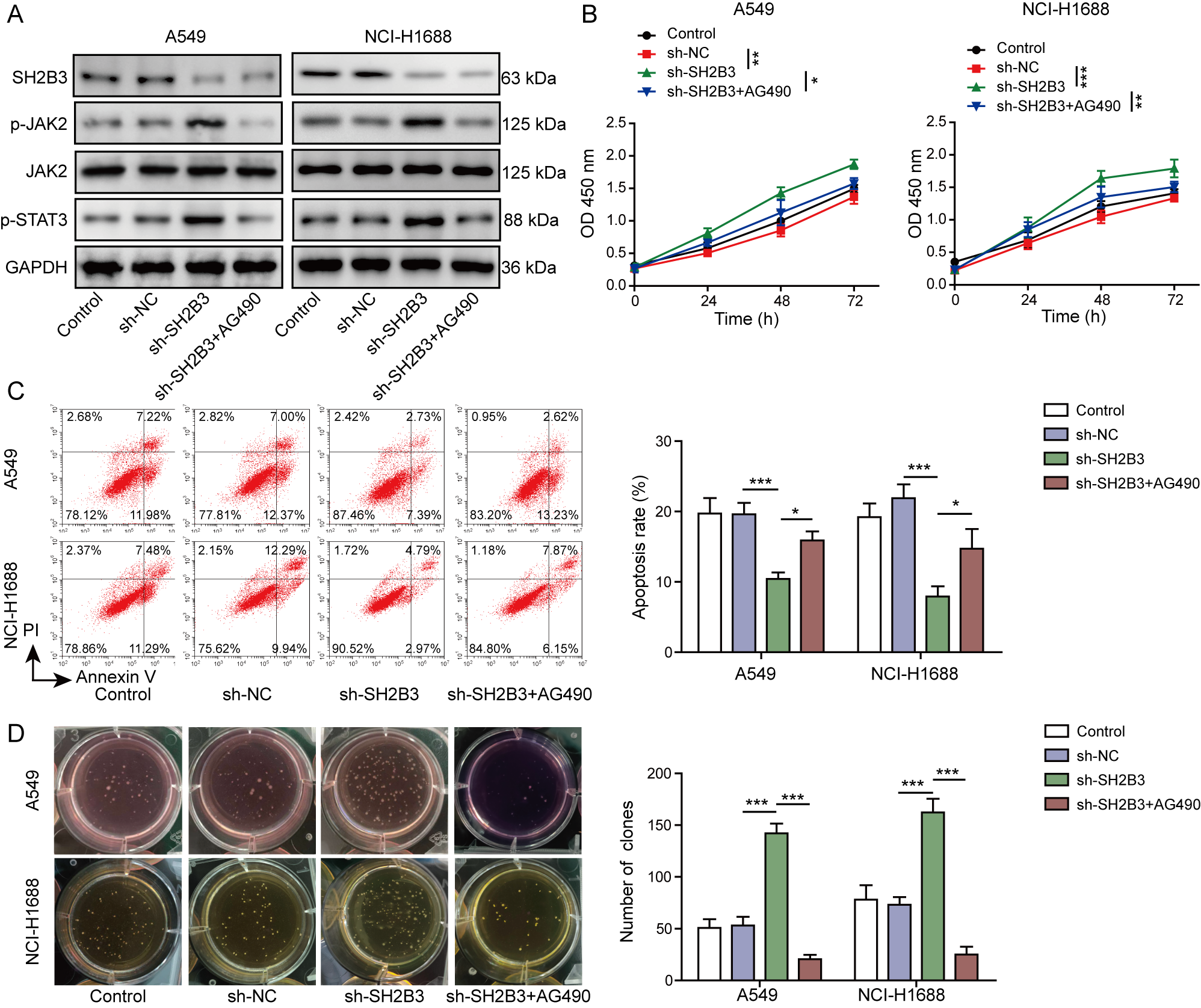

Supplement: Supplementary file 7 — Supplementary Fig.5 [file 41419_2022_4890_MOESM7_ESM.tif]

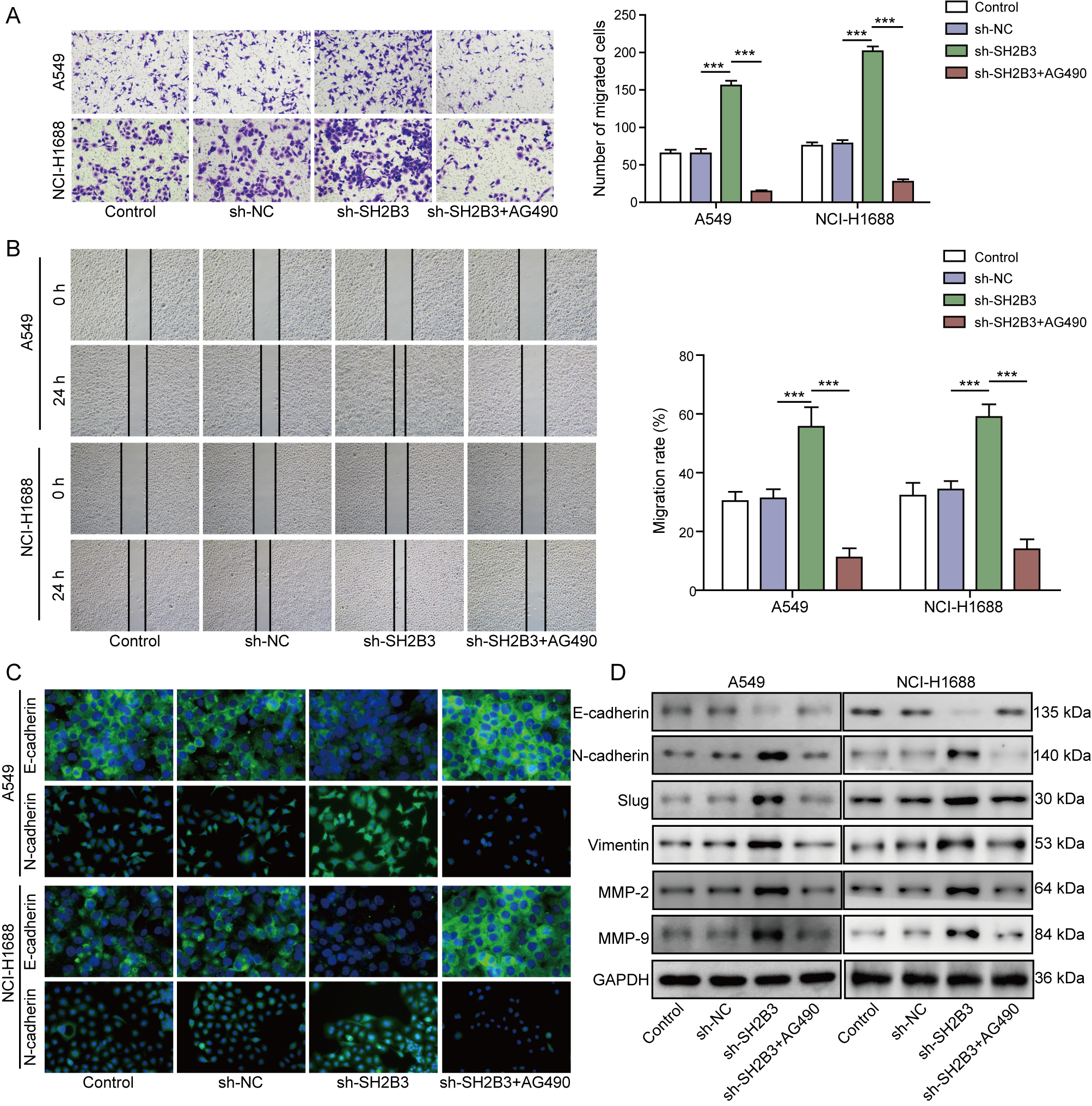

Supplement: Supplementary file 8 — Supplementary Fig.6 [file 41419_2022_4890_MOESM8_ESM.tif]

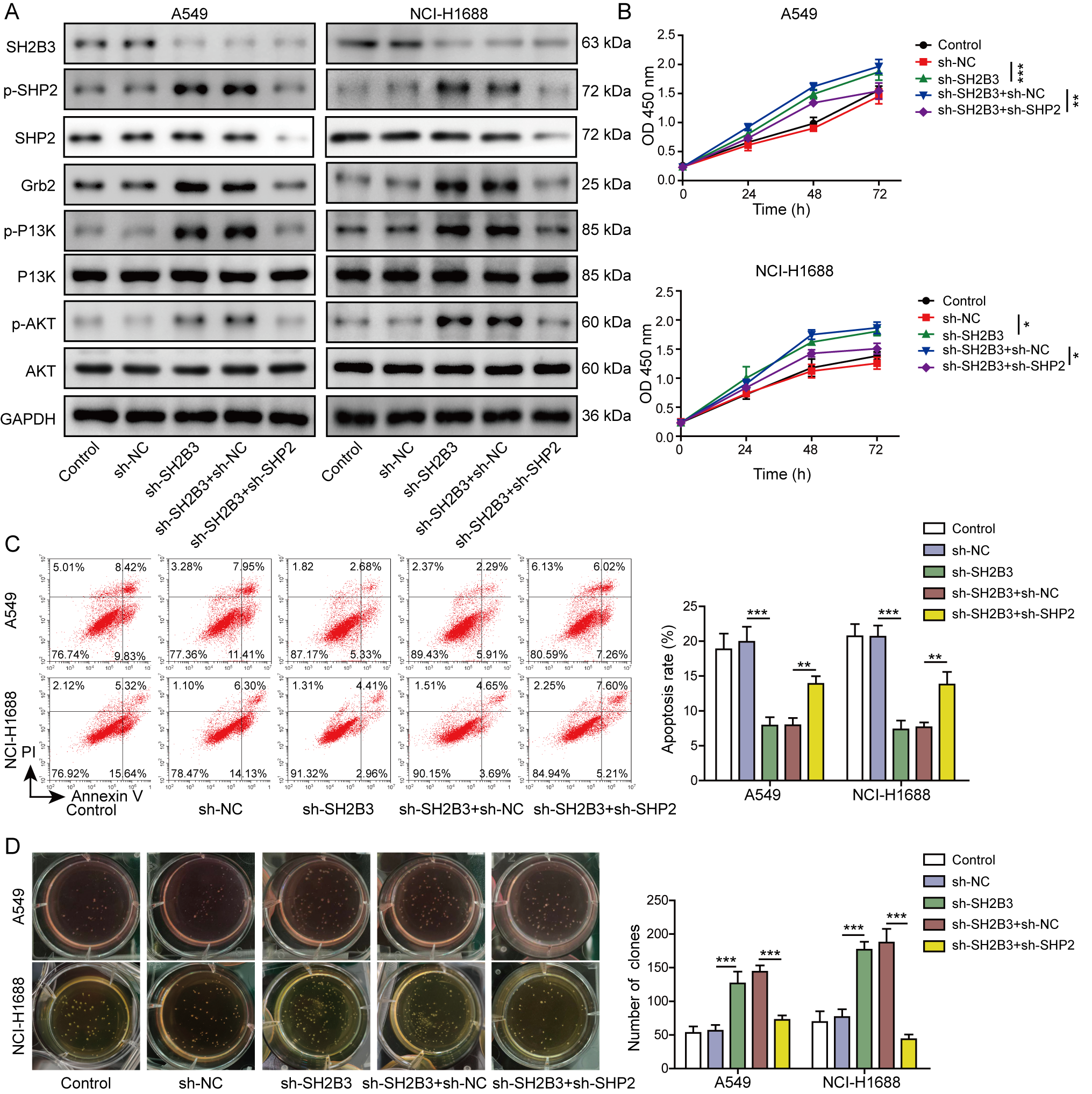

Supplement: Supplementary file 9 — Supplementary Fig.7 [file 41419_2022_4890_MOESM9_ESM.tif]

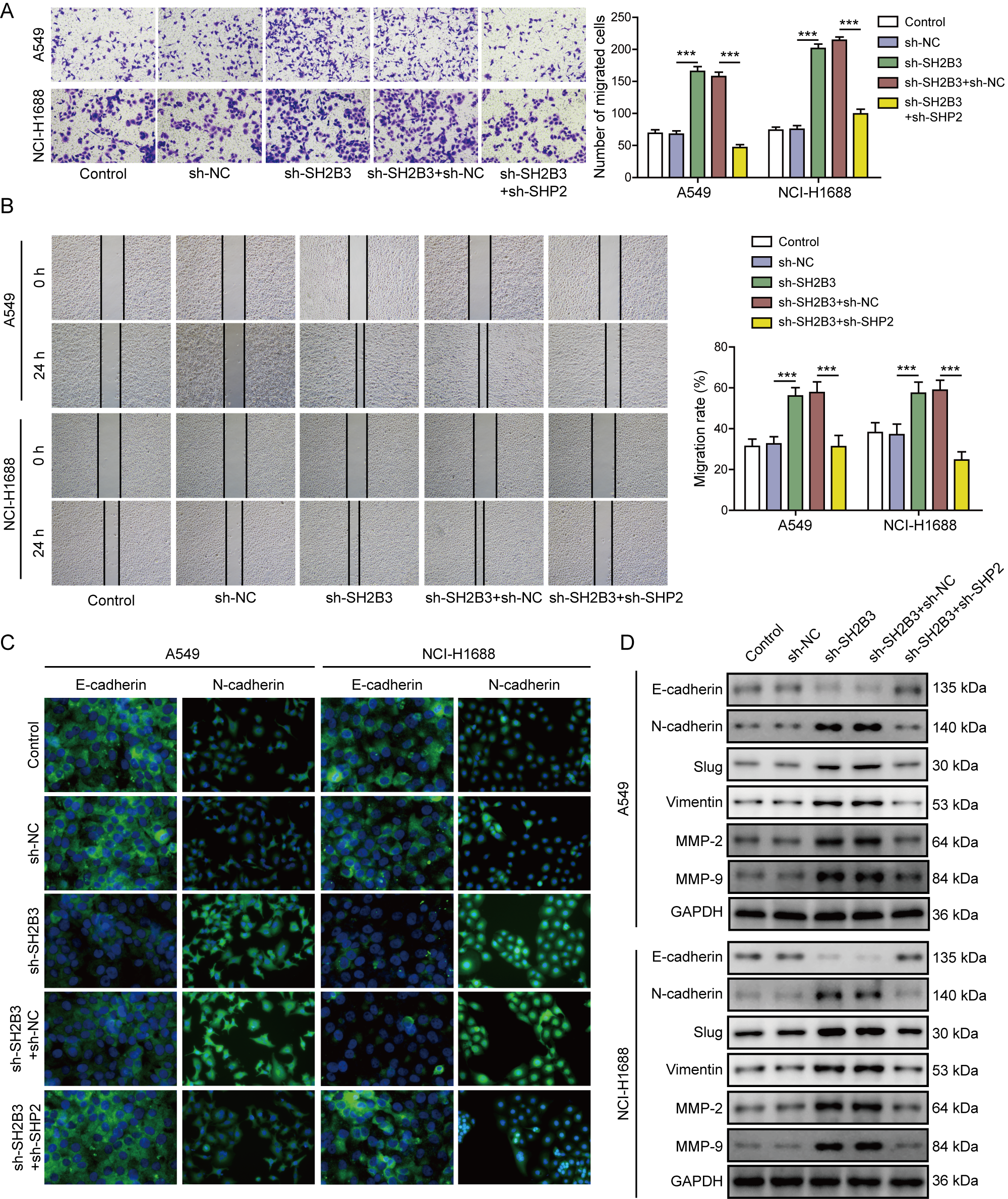

Supplement: Supplementary file 10 — Supplementary Fig.8 [file 41419_2022_4890_MOESM10_ESM.tif]
