# Supplementary material for: TGF-β1/SH2B3 axis regulates anoikis resistance and EMT of lung cancer cells by modulating JAK2/STAT3 and SHP2/Grb2 signaling pathways
Source: Cell Death Dis. 2022 May 19;13(5):472. doi: 10.1038/s41419-022-04890-x (PMC9120066; doi:10.1038/s41419-022-04890-x)
Supplement: Supplementary file 4 — Supplementary figure legends [file 41419_2022_4890_MOESM4_ESM.docx]

**Supplementary figure legends**

**Supplementary figure 1. SH2B3 promoted anoikis but suppressed proliferation, migration, invasion and EMT of Calu-1 cells.**

(A) qRT-PCR analysis of SH2B3 mRNA levels in transfected Calu-1 cells. (B) Western blotting to examine SH2B3 protein levels in transfected Calu-1 cells. (C) Flow cytometry to measure anoikis of transfected Calu-1 cells. (D) Soft colony formation to assess the number of anoikis resistance of transfected Calu-1 cells. (E) CCK-8 assay to evaluate proliferation rate of transfected Calu-1 cells. (F) Transwell assay to measure invasion capacity of transfected Calu-1 cells. (G) Scratch wound healing assay to determine the migration ability of transfected Calu-1 cells. (H) Immunostaining to test E-cadherin and N-cadherin protein levels in transfected Calu-1 cells. (I) Western blotting to measure levels of EMT-related markers including E-cadherin, N-cadherin, slug, vimentin, MMP-2, and MMP-9 in transfected Calu-1 cells. *** *p*< 0.001, ** *p*< 0.01 and * *p*< 0.05.

**Supplementary figure 2. TGF-β1 regulated anoikis and cell proliferation of lung cancer via SH2B3.**

(A) qRT-PCR analysis of SH2B3 mRNA levels in transfected cells following TGF-β1 treatment. (B) Western blotting to examine SH2B3 protein levels in transfected cells following TGF-β1 treatment. (C) Flow cytometry to measure anoikis of transfected cancer cells following TGF-β1 treatment. (D) CCK-8 assay to evaluate proliferation rate of transfected cancer cells following TGF-β1 treatment. *** *p*< 0.001, ** *p*< 0.01 and * *p*< 0.05.

**Supplementary figure 3. TGF-β1 regulated cell migration, invasion and EMT of lung cancer via SH2B3.**

(A) Transwell assay to measure invasion capacity of transfected cancer cells following TGF-β1 treatment. (B) Scratch wound healing assay to determine the migration ability of transfected cancer cells following TGF-β1 treatment. (C) Immunostaining to measure E-cadherin and N-cadherin protein levels in transfected cancer cells following TGF-β1 treatment. (D) Western blotting to measure levels of EMT-related proteins including E-cadherin, N-cadherin, slug, vimentin, MMP-2, and MMP-9 in transfected cancer cells following TGF-β1 treatment. (E) qRT-PCR analysis of TGF-β1 mRNA levels in human lung cancer tissues (n=40). (F) Survival rate in lung patients with high or low TGF-β1 levels from GEPIA database. *** *p*< 0.001.

**Supplementary figure 4. TGF-β1 modulated JAK2/STAT3 and SHP2/Grb2/PI3K/AKT signaling pathways via SH2B3.**

(A) Western blotting to determine protein levels of p-JAK2, JAK2, p-STAT3, and STAT3 in transfected cancer cells following TGF-β1 treatment. (B) Western blotting to determine protein levels of p-SHP2, SHP2, Grb2, p-PI3K, PI3K, p-AKT, and AKT in transfected cancer cells following TGF-β1 treatment. *** *p*< 0.001, ** *p*< 0.01 and * *p*< 0.05.

**Supplementary figure 5. SH2B3 regulated anoikis and cell proliferation of lung cancer via JAK2/STAT3 signaling.**

(A) Western blotting to measure protein levels of SH2B3, p-JAK2, JAK2, p-STAT3, and STAT3 in transfected cancer cells with or without AG490 treatment. (B) CCK-8 assay to evaluate proliferation rate of transfected cancer cells with or without AG490 treatment. (C) Flow cytometry to measure anoikis of transfected cancer cells with or without AG490 treatment. (D) Soft colony formation to assess the number of anoikis resistance of transfected cancer cells with or without AG490 treatment. *** *p*< 0.001, ** *p*< 0.01 and * *p*< 0.05.

**Supplementary figure 6. SH2B3 regulated cell migration, invasion and EMT of lung cancer via JAK2/STAT3 signaling.**

(A) Transwell assay to measure invasion capacity of transfected cancer cells with or without AG490 treatment. (B) Scratch wound healing assay to determine the migration ability of transfected cancer cells with or without AG490 treatment. (C) Immunostaining to measure E-cadherin and N-cadherin protein levels in transfected cancer cells with or without AG490 treatment. (D) Western blotting to measure levels of EMT-related proteins including E-cadherin, N-cadherin, slug, vimentin, MMP-2, and MMP-9 in transfected cancer cells with or without AG490 treatment. *** *p*< 0.001.

**Supplementary figure 7. SH2B3 regulated anoikis and cell proliferation of lung cancer via SHP2/Grb2/PI3K/AKT signaling.**

(A) Western blotting to measure protein levels of p-SHP2, SHP2, Grb2, p-PI3K, PI3K, p-AKT, and AKT in transfected cancer cells. (B) CCK-8 assay to evaluate proliferation rate of transfected cancer cells. (C) Flow cytometry to measure anoikis of transfected cancer cells. (D) Soft colony formation to assess the number of anoikis resistance of transfected cancer cells. *** *p*< 0.001, ** *p*< 0.01 and * *p*< 0.05.

**Supplementary figure 8. SH2B3 regulated EMT and cell migration and invasion of lung cancer via SHP2/Grb2/PI3K/AKT signaling.**

(A) Transwell assay to measure invasion capacity of transfected cancer cells. (B) Scratch wound healing assay to determine the migration ability of transfected cancer cells. (C) Immunostaining to measure E-cadherin and N-cadherin protein levels in transfected cancer cells. (D) Western blotting to measure levels of EMT-related proteins including E-cadherin, N-cadherin, slug, vimentin, MMP-2, and MMP-9 in transfected cancer cells. *** *p*< 0.001.
