# Supplementary material for: TGF-β1/SH2B3 axis regulates anoikis resistance and EMT of lung cancer cells by modulating JAK2/STAT3 and SHP2/Grb2 signaling pathways
Source: Cell Death Dis. 2022 May 19;13(5):472. doi: 10.1038/s41419-022-04890-x (PMC9120066; doi:10.1038/s41419-022-04890-x)
Supplement: Supplementary file 5 — Supplementary table 1 [file 41419_2022_4890_MOESM5_ESM.docx]

**Table 1. The correlation between SH2B3 expression and clinicopathological characteristics in lung cancer patients.**

| **Clinicopathological characteristics** | **Cases (n)** | **SH2B3 expression** | | ***P* value** |
| --- | --- | --- | --- | --- |
|  |  | **High** | **Low** |  |
| Age (years) |  |  |  |  |
| >60 | 23 | 8 | 15 | 0.0536 |
| ≤60 | 17 | 12 | 5 |  |
| Gender |  |  |  |  |
| Female | 12 | 9 | 3 | 0.0824 |
| Male | 28 | 11 | 17 |  |
| Tumor size (cm) |  |  |  |  |
| >5 cm | 19 | 11 | 8 | 0.5273 |
| ≤5 cm | 21 | 9 | 12 |  |
| Lymph node metastasis |  |  |  |  |
| Yes | 22 | 6 | 16 | 0.0036 |
| No | 18 | 14 | 4 |  |
| Smoking status |  |  |  |  |
| Yes | 16 | 10 | 6 | 0.3332 |
| No | 24 | 10 | 14 |  |
| TNM stage |  |  |  |  |
| I-II | 15 | 12 | 3 | 0.0079 |
| III-IV | 25 | 8 | 17 |  |
